# Education Research: The Future of Child Neurology Residency Training: The Perspective of a Child Neurology Society Task Force

**DOI:** 10.1212/NE9.0000000000200331

**Published:** 2026-06-12

**Authors:** Bruce H. Cohen, Donald L. Gilbert, Kathryn Xixis, Brenda L. Banwell, Azara Singh, Lindsay M. Pagano, Amy Brooks-Kayal, Laura Kirkpatrick, Nina F. Schor, Soe S. Mar, Daniel Crowder, Monique Terrell, Peter B. Kang

**Affiliations:** 1 Rebecca D. Considine Research Institute and Department of Pediatrics, Akron Children's Hospital, OH;; 2 Division of Neurology, Cincinnati Children's Hospital Medical Center, Department of Pediatrics, University of Cincinnati College of Medicine, OH;; 3 University of Virginia, Charlottesville;; 4 Johns Hopkins University, Baltimore, MD;; 5 University of Wisconsin-Madison;; 6 Vanderbilt University Medical Center, Nashville, TN;; 7 Department of Neurology, University of California Davis School of Medicine, Sacramento;; 8 University of Pittsburgh, PA;; 9 University of Rochester, NY;; 10 Washington University in St. Louis, MO;; 11 Oregon Health and Science University, Portland;; 12 Child Neurology Society, Roseville, MN; and; 13 Department of Neurology, University of Minnesota Medical School, Minneapolis.

## Abstract

**Background and Objectives:**

Postgraduate residency in the United States is complex, and each medical field faces unique educational challenges. Child neurology has had a distinct identity for decades, yet its training curriculum originated from a joint venture of 2 larger and older fields, pediatrics and neurology. The traditional five-year training sequence consists of 2 years of pediatrics, 1 year of adult neurology, and 2 years of child neurology, as well as all other educational experiences, including electives. The contents of each major component have changed, in some instances dramatically, over the years, yet the original basic structure remains.

**Methods:**

In anticipation of upcoming reviews of the child neurology training curriculum, the Child Neurology Society (CNS) convened a task force to review key aspects of this training curriculum. The task force members were surveyed anonymously both before and after a series of discussions to consider the current and expected educational needs of child neurologists in the mid-21st century, following a modified Delphi approach.

**Results:**

The consensus was that child neurology has matured immensely as a field, with substantial subspecialization becoming common at major academic medical centers. There was significant variability in the availability of pediatric neurologic subspecialty training in fields such as neurogenetics and neuromuscular neurology across the United States. The preponderant view of the Task Force was that the child neurology training curriculum should be reviewed and potentially modified in accordance with the evolving educational needs of child neurologists, particularly with regard to general pediatrics and adult neurology training. The spectrum of faculty expertise and curricular emphasis across programs, viewed as a strength of training options, precluded reaching consensus on details of appropriate changes.

**Discussion:**

The findings of the task force indicate that there will be a need for ongoing evaluation and updating of the child neurology training curriculum in future years. Before the next formal review of the child neurology training curriculum by the Accreditation Council for Graduate Medical Education, we recommend a robust discussion among multiple stakeholders, including accreditation bodies, medical specialty boards, and professional societies such as the CNS that represent child neurologists in practice and in training.

## Introduction

As medical knowledge expands and clinical practice evolves, requirements for training medical residents for competent practice and board certification also change. This process of periodic review and revision has emerged again for the specialty of child neurology in the United States and, as was the case during the last iteration in 2014, controversy can be expected.

Dedicated postgraduate training in child neurology is recognized by the Accreditation Council for Graduate Medical Education (ACGME), which came into existence in 1981 and oversees board accreditation activity of residencies and fellowships. The American Board of Psychiatry and Neurology (ABPN) has issued certifications for the subspecialty of “Neurology with Special Qualification in Child Neurology” since 1969.^1,2^

As with all specialties in medicine, child neurology has advanced in ways that were unimaginable in the 1970s. For example, magnetic resonance imaging and other modalities have replaced pneumoencephalograms^3^ and next-generation sequencing has replaced karyotypes and single-gene testing.^4^ In addition, an ever-proliferating armamentarium of small-molecule drugs now shares the therapeutic stage with enzyme replacement therapies, antisense oligonucleotides, and gene replacement therapies.^5^ In fact, in many areas of child neurology, the rapid expansion of diagnostic and therapeutic knowledge has reached a level where subspecialty fellowship training has become a common, although not mandatory, choice among trainees.

Given these changes and the routinely scheduled re-evaluation of training requirements by the ACGME, it is worth asking whether the current curriculum for child neurology residency has the flexibility to accommodate the evolving educational needs of child neurologists who will be practicing well into the mid-21st century. In addition, the cost of training, both in terms of financing the salaries of those in training and opportunity costs associated with lower resident salaries and deferred debt repayment by the trainees, is an order of magnitude different now than it was 50 years ago. These changes create a conundrum because neither time nor funds ever seem sufficient to impart the totality of pertinent knowledge to child neurologists during their training. Furthermore, the knowledge base required for effective practice in child neurology continues to expand.

For half a century, the standard and most common training pathway for child neurologists consisted of 2 years of general pediatrics and 3 years of neurology (1 year of adult neurology followed by 2 years of child neurology), qualifying the graduate to sit for board certification examinations in both General Pediatrics, through the American Board of Pediatrics (ABP), and Neurology with Special Qualification in Child Neurology (NwSQiCN), through the ABPN. At traditional academic institutions, these graduates mostly practice in divisions housed in either a Department of Pediatrics or a Department of Neurology.^6^ Those holding board certifications from the ABP and/or ABPN have filled senior leadership roles, including chair positions, in the corresponding departments. Since the advent of child neurology, the field exists as a pediatric subspecialty, but not specifically an ABP subspecialty, as the board granting the child neurology certification is the ABPN. In contrast to many other nonsurgical pediatric subspecialties, the ABPN NwSQiCN certification implies competence to care for both children and adults, within the context of specific residency training, continued clinical practice, and continuing medical education. As we plan next steps in child neurology training, it will be vital to balance both the evolving landscape of child neurology practice and the importance of providing for the needs of the increasing number of children with chronic neurologic disorders who survive well into adulthood.^7^

The last formal ACGME review and revision of Child Neurology Certification requirements were completed in 2014. In light of the anticipated next cycle of ACGME program review, now scheduled for 2027, the Child Neurology Society (CNS) formed a task force with the intent to review the current requirements for residency training in child neurology and to consider whether curricular modifications are needed to adapt to the tremendous evolution of our specialty over the decades. The issues to be discussed included but were not limited to the current and future neurologic needs of patients across the lifespan from the prenatal stage through early adulthood, the identification of gaps in the current training pathways, the duration of general pediatric training and adult neurology training for child neurologists, the total training duration, and the potential effects of any changes on the child neurology workforce.

## Methods

### Overall Structure of Task Force Activities

The Task Force conducted a survey before their first meeting and a second survey after the final meeting. Each survey invitation was followed by one reminder, for a total of 4 outreach attempts. The use of sequential surveys and structured group discussions shares characteristics with consensus-building frameworks and was designed to follow a modified Delphi approach.

### Task Force Composition and Characteristics

The CNS leadership appointed child neurologist members to the Training the Future Child Neurologist Task Force. Nine members were selected based on previous contributions to training-related initiatives; 4 additional members were selected through an open application process available to all CNS members. Applicants were asked to submit curriculum vitae and responses to the following 4 questions: (1) What size program do you currently or did you formally manage? (2) Are you a current or former Program Director? (3) What is your primary practice type? (4) What year did you complete residency? The 4 additional task force members were selected from 65 applicants, with the goal of ensuring representation across the spectrum of child neurology practice based on responses to the questions and credentials. The final Task Force included 13 CNS members and, ex officio, the Chief Executive Officer/Executive Director (CEO/ED) of the CNS as a nonvoting member. All but one member of the Task Force are co-authors of this article, reflecting their full participation in the development, discussion, and interpretation of the group's work. One member of the task force recused themselves from the article author list because of a conflict of interest that arose after completion of the task force activities but before publication. Although the CEO/ED contributed actively to the Task Force deliberations, she did not participate in the survey component of the process, in keeping with her nonclinical, administrative role.

The composition of the Task Force reflected diversity in career stage, program size, geographic location, and practice type. Among the 13 CNS-appointed members (excluding the CEO/ED), 9 were female, 10 were White, and 12 held US board certification through the American Board of Psychiatry and Neurology (initial certification years ranging from 1988 to 2022), while 1 was certified through the Royal College of Physicians and Surgeons of Canada. The mean time since initial certification was 14 years (range 2–36 years), and 12 members were currently clinically active in academic or hospital-based practices.

Specialty certifications among the group included Neurodevelopmental Disabilities (1), Epilepsy/Clinical Neurophysiology (3), and Neuromuscular Neurology (1). Many members held current or past leadership positions, including serving on the ABPN Board of Directors (2), as CNS officers (4), and as leaders within the Professors and Educators of Child Neurology (3) or AAN Child Neurology Section (1). Regarding institutional leadership, 5 members had served as current or former division or department chairs. A total of 8 members held roles as current or former residency program directors.

### Initial Task Force Member Survey

The 2023 ACGME program requirements were compiled,^8^ and 2 members of the Task Force (the Task Force Chair, BHC, and DLG, an experienced child neurology educator) independently selected the requirements they believed to be the most pertinent for discussion. Their selections were nearly identical. An initial survey was emailed to the 13 CNS members to assess responses to 24 statements reflecting these selected requirements.

Two statements, one from the ACGME program requirements and one from the ABPN, addressed the definition of a child neurologist. We included 22 other statements from the 2022 ACGME Child Neurology Program Requirements (eTable 1). These included 4 statements about institutional requirements, 3 about time-based requirements in pediatrics (pathways), 3 about educational curriculum, 4 about time-based requirements in adult neurology (1 general, 3 specific), and 8 about clinical training in child neurology (including 1 for child psychiatry). The items were structured on a 7-point Likert scale (strongly disagree to strongly agree), with opportunities for free-text comments. To better assess directional agreement or disagreement, responses marked “neither agree nor disagree” were excluded from analysis; this decision was made prospectively by the authors before data review. “Consensus” was defined as ≥75% of *respondents* (not all eligible task force members) either agreeing or strongly agreeing—or disagreeing or strongly disagreeing—with a statement. For the initial survey, this threshold was applied to the 9 members who responded (9/13; 69%). While this met our predefined analytical threshold, we acknowledge that applying a consensus criterion to such a small subgroup may limit interpretability, and results from the initial survey were used primarily to guide structured discussions.

### Task Force Meetings and Repeat Survey

Seven virtual meetings were convened from July 2023 through January 2024; each meeting lasted approximately 1 hour. The training requirements and initial survey results were discussed in detail. Additional input was obtained from the ABPN President and CEO, the senior child neurologist member of the ACGME Neurology Review Committee, and the current 2 leaders of the Child Neurology Residency Program Directors Forum. The majority of discussion focused on historically divergent areas—particularly time-based training requirements in pediatrics and adult neurology. After the final task force meeting, a repeat survey was conducted that included the original 24 items plus 2 new items addressing neurogenetics and molecular therapies.

### Standard Protocol Approvals, Registrations, and Participant Consents

The CNS determined that this study did not constitute human subjects research because we gathered program evaluation data rather than human subjects data. Thus, we did not seek ethical approval. No patients were involved in this study.

### Data Availability

All data pertinent to the study are presented in this article and supplemental materials.

## Results

### Survey

Nine of 13 CNS Task Force members completed all 24 items on the initial survey, and 12 members completed the 26 items on the follow-up survey (eTable 2). None of those who completed one or both of the surveys omitted any of the questions. Based on our definition of consensus as noted in Methods, we divided responses in areas of consensus vs areas of nonconsensus (Tables 1 and 2).

**Table 1:** Areas of Consensus From Final Survey Results

Child neurology involves the diagnosis, evaluation, and management of, and the advocacy for, infants, children, and adolescents with either primary or secondary disorders of peripheral and central nervous systems
Neurology with special qualification in child neurology is a specialty that involves the specialization in neurology with particular skills in diagnosis and treatment of neurologic disorders of the neonatal period, infancy, early childhood, and adolescence
The Sponsoring Institution or participating sites must also sponsor ACGME-accredited residency programs in pediatrics and neurology
There must be a program letter of agreement (PLA) between the program and each participating site
There must be a program coordinator provided with dedicated time and support adequate for administration of the program based on its size and configuration
The program must contain a broad range of structured didactic activities; residents must be provided with protected time to participate in core didactic activities
The program must include advancement of residents' knowledge of ethical principles foundational to medical professionalism
The program must cover advancement in the residents' knowledge of the basic principles of scientific inquiry, including how research is designed, conducted, evaluated, explained to patients, and applied to patient care
The curriculum must be organized to provide at least 12 FTE months of clinical child neurology
The 12 FTE months of clinical child neurology must include at least 4 FTE months of outpatient experience
The curriculum must provide at least a one-month FTE experience under the supervision of a qualified child and adolescent psychiatrist
The curriculum must provide 3 months elective time with assignments that accommodate individual resident interests and previous education
Training must include management responsibility for hospitalized patients with neurologic disorders, including pediatric patients with acute neurologic disorders, in an intensive care unit and an emergency department
Training must include experience in the evaluation and management of patients with disorders of the nervous system requiring surgical management
Training must include assignment on a consultation service to the medical, surgical, and psychiatric services
Residents must attend a longitudinal/continuity clinic at least one half-day weekly throughout the duration of the program
Neurogenetics should be a mandatory part of training (or child neurology training)
Although not necessarily available at all training sites, delivery of new therapies, including molecular therapies (e.g., oligonucleotides and gene therapy), should be at least part of the didactic training experience

**Table 2:** Areas of Nonconsensus From Final Survey Results

Child neurology education must be conducted in centers where there is active ongoing research in both clinical and basic neuroscience fields
Before appointment in the child neurology program, residents must have successfully completed 2 years of education in pediatrics
Before appointment in the child neurology program, residents must have successfully completed 1 year of education in pediatrics and one year of training in adult medicine (internal medicine or family medicine)
Before appointment in the child neurology program, residents must have successfully completed 1 year of education in pediatrics and 1 year of education in family medicine or internal medicine
The curriculum must be organized to provide at least 12 FTE months of adult neurology under the supervision of faculty members certified by the ABPN or AOBPN in neurology
The 12 FTE months of adult neurology must include 6 mo on inpatient (defined as requiring more than 50 percent of time managing patients admitted to an inpatient service requiring neurologic care) rotations
The 12 FTE months of adult neurology must include 3 mo of outpatient (defined as requiring more than 50 percent of time spent managing patients in an outpatient clinic setting) clinical adult neurology rotations
The 12 FTE months of adult neurology must include 3 mo of elective adult neurology clinical experiences. Rotations on subspecialty areas of neurology, including neuroradiology, neuropathology, and neurophysiology, may be counted toward this requirement

### Areas of Consensus

The Task Force largely demonstrated consensus on general institutional settings needed for child neurology residency programs, program coordinator support, importance of instruction in ethics and research, and key aspects of the child neurology components of training programs, such as time-based requirements in continuity clinic, outpatient services, and inpatient services. There was also a high level of agreement about inclusion of genetics and molecular therapeutics within the child neurology curriculum. The advances in digital education have made such topics increasingly accessible, which may benefit smaller programs with less access to internal resources.

### Areas Lacking Consensus

Despite recurring and intensive discussions, the Task Force did not reach consensus on 2 pivotal issues, the length and structure of preliminary postgraduate training before the neurology years and the length and composition of adult neurology training within the neurology years. The 2014 revised requirements, which added specificity to the requirements for the adult neurology year, did not fare any better. Most respondents disagreed with the inpatient/hospital-based requirement duration, and there was limited support for the current outpatient and elective (adult neuropathology, EEG, neuroimaging) requirements.

## Discussion

The Child Neurology Task Force reviewed training requirements pertinent to the anticipated review of training requirements by the ACGME and ABPN in 2027. At onset, consensus was present for only 58% of current requirements reviewed. After information gathering and a series of discussions during virtual meetings, a modest increase, to 69%, emerged. This suggests that, within national leadership of child neurologists, there are substantial disagreements with at least some requirements and in assessments of how well current training practices and certification requirements serve our trainees and patients. Consensus was not reached on several key training questions, including pediatric prerequisite duration and adult neurology exposure. This lack of agreement, although a limitation, also reflected the complexity of these issues and reinforced the need for broader, more inclusive dialog in future efforts. Using the 75% consensus threshold as a guide, it was thus not possible for this Task Force to make a clear singular recommendation for changes to either the general pediatrics or adult neurology components of child neurology residency training. Notably, however, although the Task Force did not reach consensus criteria on the general pediatrics or adult neurology time-based requirements, the results indicated that certain issues merit further deliberation. For example, the majority of Task Force member opinions aligned with the results of single–time point surveys showing that a majority of child neurology residency program directors,^9^ as well as child neurologists in practice^10,11^ and in training,^10^ favor reducing the specific time requirements spent in adult training (e.g., the adult “year”) and also did not believe that child neurology residents need to complete 2 years of education in pediatrics.

The lack of consensus regarding the duration of both pediatric and adult neurology training likely reflected child neurology's origins at the crossroads of the 2 larger fields of neurology and pediatrics. It is exceedingly rare for an academic child neurology unit to be a separate department at a university in the United States. Child Neurology divisions reside variously within Departments of Neurology at some institutions and Departments of Pediatrics at others. This may lead to greater institutional variability in the prioritization of certain aspects of training. Finally, as has been noted above, massive changes have occurred in clinical neurology practice and neuroscience knowledge, and the mismatch between these changes and our 50-year-old training structure likely manifested differently based on individual experiences and subspecialty demands.

Given that a recent survey showed that only 3% of child neurologists provide general adult neurology services,^11^ some have suggested that a full year of adult training need not be offered by all programs, and that, for many, a shorter period of adult training would be adequate.^12-14^ As the field continues to evolve and as transitional care models improve, it may be appropriate to explore whether a more targeted or abbreviated adult neurology experience could achieve the same educational and clinical goals. Nevertheless, a foundational understanding of adult neurology remains vital for ensuring that child neurologists are prepared to manage the full continuum of neurologic disease. Furthermore, given rapid advancements in neurology knowledge, the foundation for adapting and changing practice from children to adults or vice versa gained in residency may need to be heavily supplemented by continuing education. It has been proposed that program requirements could allow for more latitude regarding the length of adult neurology training, and this is an option that could be discussed with the ACGME and the ABPN. This structure would allow those programs to provide more child neurology-focused learning opportunities, longitudinal patient care, supervision, mentorship, and subspecialty exposure starting in the first year. However, this would also require either modification of ABPN certification requirements or a separate certification designation from NwSQiCN. By contrast, other institutions may determine that focusing on the fundamentals of the neurologic examination and principles of localization in the nervous system in adult neurology during the first year continues to be the optimal training for their child neurology residents.^15^ Adult neurology training in child neurology residencies has also been valued for its contribution to clinical skills, particularly in stroke, neuro-ophthalmologic emergencies, and other acute neurologic presentations, which occur at higher volumes in adults and provide rich opportunities to develop strong localization skills. Building proficiency in neurologic localization remains a recognized area of relative weakness in child neurology training and is addressed, in part, through these adult neurology rotations. Medical students could reflect on these options during the match process and determine which type of program best fits their interests. Specifically, providing comprehensive subspecialty experiences during residency training could be institution specific: some programs may have sufficient expertise in most or all subspecialties, with less of a need for the trainees to rotate on adult neurology; other programs may use more adult or life-span (adult and pediatric) clinical learning environments to provide training in some subspecialty areas.

A recent study of another group of child neurologists, using a structured, Delphi methodology with anonymous iterative feedback and voting, resulted in a consensus for a mean duration of training (including pediatric training) of 46.5 months that included 12.5 total months of “traditional” pediatric rotations, 3 months of inpatient adult neurology, and 2 months of outpatient adult neurology, with “program-level flexibility” for the remaining rotations that would include topics in child neurology and electives.^16^

As we consider opportunities to modernize child neurology training, it is important to appreciate the needs of the increasing number of children with chronic neurologic disorders who survive well into adulthood. It is vital that neurologists of all backgrounds, whether their training was focused on adult neurology or child neurology, strive to ensure that children with neurologic disorders continue to receive high quality care throughout their lifespan. The current challenges with care transitions may be amplified by the asymmetry of cross training, with only 3 months of child neurology training for adult neurologists. An emphasis on the care of young adults and transitions to adult neurology could be enhanced as part of adult neurology training for child neurology residents and child neurology training for adult neurology residents.^7^ An example is the need to enhance counseling regarding reproductive health in the setting of epilepsy, which spans both pediatric and adult age groups.^17^ The responsibility of effective training allowing for transition of care lies with both the child and adult components of the training programs.^7^

Many pediatric subspecialists care for patients who ultimately require transition to adult providers, yet they do so without formal adult training. Competency in general neurology for both children and adults may prove especially useful in underserved rural areas. Furthermore, some child neurologists, both independent as well as in university-based practices, provide subspecialty care well into adulthood, typically after formal fellowship training.

Previous studies have documented ongoing challenges regarding the child neurology workforce^18-20^ and clinical revenues.^21^ Ideally, any changes to the child neurology training curriculum would be at worst neutral and, at best, would bolster the child neurology workforce with regard to numbers, scope of practice, clinical revenue generation, and research revenue capture. The increasing popularity of hospitalist models of care delivery in some pediatric institutions also has implications for the child neurology workforce, with new career options but also new challenges involving workloads and the allocation of financial credit for the work involved.^22^ Pediatric neurocritical care has emerged as a newer field that is rapidly maturing and yielding new educational opportunities and needs for child neurology residents.^23^

Funding for the current most common training pathway (2 years of pediatric residency followed by 3 years of neurology training) will need to be considered. Current mechanisms for funding residency positions differ significantly among training programs.^9^ Those training programs established years ago may be able to fund all 5 years through government sources, while new programs or those with expanded numbers of trainees may depend on institutional support for some or all of the training costs. The salaries of residents often comprise an ill-defined patchwork of funds derived from many sources, supported by institutional agreements built over many years. A financial equipoise exists at each institution and agreements between the Graduate Medical Education office and departments are often based on the time dedicated to pediatrics, adult neurology, and child neurology service time. If major training changes occur, such as eliminating a year of pediatric training or decreasing the mandatory time required for adult neurology training, changes in current agreements for funding resident salaries may be challenging for some institutions.^9^

Other possible positive and negative consequences of changing the training model were discussed. It was proposed by some members of this task force that shortening the total training time by a year and shortening the training in adult neurology in exchange for more (perhaps subspecialty) training in child neurology will attract more medical students to train in child neurology. Others noted that, while a small minority of child neurologists care for significant volumes of adults, the flexibility to do so may broaden some career opportunities, particularly with respect to evolving care models for young adults with childhood-onset complex neurologic disorders. Although not applicable for the vast majority of working child neurologists, for those who aspire to higher level leadership positions in academic medicine, child neurologists who have become Chairs of Pediatric and Neurology Departments serve as role models. There is not a uniform path for advancement, so child neurologists who aspire to those positions must consider both practice settings and their home academic departments when deciding on which certification(s) to obtain and maintain. Changes in training curricula should not introduce new barriers to academic career advancement for child neurologists.

After the deliberations of the Task Force were completed, the ABPN convened a Crucial Issues Forum in April 2025 that discussed the potential to transition adult and child neurology training to a competency-based medical education (CBME) model, following the lead of other specialties and graduate medical educational systems in place in other countries.^24^ This approach may yield yet another option for modernizing child neurology training. Child neurology has taken steps toward CBME already, with the adoption of educational milestones in 2015, followed by revisions.^25^

Our study had several important limitations. The initial survey achieved a 69% response rate (9 of 13 eligible Task Force members), while the second had 92% participation (12 of 13), despite repeated outreach. Owing to the anonymous nature of the surveys, it was not possible to send personalized reminders to nonrespondents, nor to discern the reasons for nonparticipation. The small sample size and incomplete participation in the first round limited the generalizability and robustness of the findings. Although we applied a 75% agreement threshold to define consensus, this was calculated based on the number of actual respondents rather than the total eligible membership. Applying this threshold to a group of 9 increased susceptibility to selection bias and may have led to overstatements regarding the presence or absence of consensus during that phase. The initial survey was primarily intended to guide discussion rather than serve as a standalone outcome measure.

The survey instrument was developed specifically for this initiative and was not externally validated. Neutral (“neither agree nor disagree”) responses were excluded from quantitative analysis by previous decision, which may have influenced response distribution. No statistician was involved in survey design or analysis because the study did not involve formal statistical testing. In retrospect, certain items in the survey may have been difficult to answer based on responses to other items.

The composition of the Task Force may have also introduced bias. Ten of the 13 members were senior academic leaders—department chairs, division chiefs, or residency program directors—and many held national leadership roles within the CNS, AAN, or ABPN. While this brought institutional insight, it may have constrained openness to transformational change. The absence of trainees and limited representation of junior faculty may have narrowed the diversity of perspectives. Given the focus of the article—“what should training look like to prepare future child neurologists for independent practice?” we believed that it was essential to include voices who could reflect both on their training and their real-world practice needs. Nevertheless, we agreed that the perspective of trainees remains valuable and acknowledged that future initiatives would benefit from more formal incorporation of resident and trainee perspectives.

It is certain that child neurologists being trained today face an emerging landscape that is both exhilarating and challenging.^14^ They must contend with a dramatic increase in scientific and clinical knowledge across the field in the 21st century, with advances in neonatal/fetal neurology,^26,27^ molecular genetic diagnosis,^28^ neuro-oncology,^29^ and genetic therapies^30^ being examples of a broad transformation. At the same time, foundational knowledge regarding the neurologic examination,^31^ neuroanatomy, neurophysiology,^32,33^ and the clinical phenotypes of rare neurologic diseases continues to be essential for a child neurologist to interpret fetal neuroimaging and the latest genetic test reports, while in the next breath, counseling families on many issues surrounding what is often a serious illness in their children,^34^ including which novel FDA-approved therapies and which clinical trials might be most appropriate for their children, along with other resources that may be needed such as palliative care.^35^ The formats and types of educational experiences continue to expand with the ongoing development of new electronic teaching tools,^36^ including ones using large language models.^37^ For many childhood-onset neurologic diseases, the multiorgan system manifestations make it essential for child neurologists to have adequate training in general pediatrics, and the survival into adulthood makes it essential for child neurologists to have adequate training in adult neurology.

To move forward, next steps should include involving a larger group of stakeholders and consideration of the workforce issues. Organizations that should be involved in further deliberations include accreditation bodies such as the Accreditation Council for Graduate Medical Education (ACGME), medical specialty boards such as the ABPN, and professional societies such as the CNS that represent child neurologists in practice and in training. Consideration of best training practices by leaders in the field suggests that the path forward will include updates to the dedicated child neurology portion of the curriculum and may also include changes to the requirements for general pediatrics and adult neurology.

## Glossary

ABP: American Board of Pediatrics
ABPN: American Board of Psychiatry and Neurology
ACGME: Accreditation Council for Graduate Medical Education
CBME: competency-based medical education
CNS: Child Neurology Society
